# Development of a 3D‐printed phantom for total skin electron therapy dose assessment

**DOI:** 10.1002/acm2.14520

**Published:** 2024-09-16

**Authors:** Andrew Lee, Zaid Alkhatib, Mounir Ibrahim, Broderick McCallum‐Hee, Joshua Dass, Matthew Fernandez de Viana, Pejman Rowshanfarzad

**Affiliations:** ^1^ School of Physics, Mathematics and Computing The University of Western Australia Crawley WA Australia; ^2^ Department of Radiation Oncology Sir Charles Gairdner Hospital Nedlands WA Australia; ^3^ Centre for Advanced Technologies in Cancer Research (CATCR) Perth Australia

**Keywords:** 3D‐printing, dosimetry, TSET

## Abstract

**Purpose:**

Total skin electron therapy (TSET) is a complex radiotherapy technique, posing challenges in commissioning and quality assurance (QA), especially due to significant variability in patient body shapes. Previous studies have correlated dose with factors such as obesity index, height, and gender. However, current treatment planning systems cannot simulate TSET plans, necessitating heavy reliance on QA methods using standardized anthropomorphic phantoms and in‐vivo dosimetry. Given the relatively few studies on rotational techniques, comprehensive data in commissioning could streamline the process.

**Methods:**

Developing a full‐body phantom would enable a more thorough TSET commissioning process, including testing for position‐specific dose distributions and comprehensive measurements across all body surfaces, unlike the typical torso‐only phantoms. This was created using digital modeling software, fabricated using 3D‐printing FDM technology, and filled with tissue‐equivalent gelatine. The phantom was positioned at an SSD of 340 cm and irradiated with a standard rotational TSET plan using the 6E HDTSE mode on a Varian TrueBeam linac at gantry angles of ± 18° from the horizontal. The dose was measured at over 50 points across the surface using Gafchromic EBT3 film.

**Results:**

Dose distributions were generally consistent with existing literature values from in‐vivo dosimetry, with several position‐specific differences identified, including the hands and scalp compared to conventional positions. Hotspots were observed for the mid‐dorsum of the foot and nose, with areas under 80% of the dose identified as the soles of the feet, perineum, vertex of the scalp, top of the shoulder, and palm of the hand. Additionally, analysis using an interpolated dose heatmap found that 90% of the pixel area received a dose within 10% of the prescribed dose, indicating good uniformity with the commissioned technique.

**Conclusions:**

With high agreement with the current literature, a 3D‐printed phantom proves effective for measuring doses in areas typically unmeasurable in TSET commissioning.

## 1 INTRODUCTION

Total skin electron therapy (TSET), also known as Total Skin Electron Irradiation or Total Skin Electron Beam therapy, is a specialized radiotherapy technique developed for the treatment of Mycosis fungoides (MF) and Sézary Syndrome. Typically, a patient is irradiated with an electron beam at extended SSDs, treating the surface of the skin. This treatment tends to be highly effective, with high remission rates even in late‐state MF. Consequently, it stands out as a highly effective treatment option for the management of MF.^1^ However, due to the low prevalence of MF as well as technical and physical requirements, the implementation of TSET and the treatment positions used are not standardized and vary significantly between centres.^2^, ^3^ Thus, TSET is only implemented in large institutions, with low accessibility even in populated countries such as India.^4^

TSET techniques are largely broken down into three main approaches: the modified Stanford technique, rotational techniques, and lying‐down techniques. The modified Stanford technique involves six treatment positions to maximize exposed skin and is the most common treatment.^2^ The rotational technique, meanwhile, involves patients standing on a constantly rotating platform during irradiation and has the advantage of lower beam on time.^5^ Both have seen widespread usage^6^, ^7^ although lying‐down techniques are reserved for pediatric and non‐ambulatory patients.^8^, ^9^ While some studies conclude that dose uniformity is satisfactory and roughly equivalent between the two main techniques,^10^ a few recent studies show superior dose coverage with the rotational technique.^11^ Nevertheless, studies on rotational techniques, including in‐vivo dosimetry results, are fewer than those on the modified Stanford technique and involve fewer patients.^12^

This in‐vivo data is particularly important in TSET due to the high variation in dose distributions based on patient body shape. Correlations have been found for obesity, height, and gender.^13^, ^14^ This results in a wide range of doses delivered, with Antolak's study finding a maximum variation of 2%−107% the prescribed dose for the upper medial thigh of 53 patients.^13^ For areas such as the perineum and lower back, the difference was attributed to the larger SSD during rotation, while areas such as the mid medial finger had reduced penetration for all angles in thick fingers. Additionally, as the radius of curvature increases, the tangential dose is decreased, which disproportionally affects obese patients. This reduces uniformity particularly in the Stanford technique where only six positions are utilized.^11^ Despite the high variations in distributions, treatment planning in TSET often does not significantly account for patient shape or tumor progression and uses fixed field setups and treatment positions. This is due to the current inability to test these factors. Compared to typical radiotherapy techniques, treatment planning systems cannot simulate TSET due to SSDs of 3−4 m, significantly different compared to conventional radiotherapy.^15^ Combined with the low occurrence rates of MF and the resulting lack of extensive clinical data in treatment centers, treatment planning, and verification are more difficult than with regular radiotherapy techniques.

Given the relative lack of data for rotational TSET, the value of data obtained during the commissioning of the technique is significant. Typically, treatment commissioning and simulation are performed with anthropomorphic phantoms, such as the Alderson RANDO head and torso phantom.^15^ While this covers the main areas, regions with the largest variations, such as the inner thigh and perineum, are not measurable on these phantoms. Furthermore, the phantoms are inflexible and only represent a standard‐sized patient, which physically limits the ability to simulate various treatment positions and body sizes. This limitation is particularly significant for new implementations and techniques. A case study by Evans et al. on a reclined technique reported a 28% underdose compared to the expected planned dose.^8^ Of the 28%, 15% was due to the difference in patient‐specific body factors compared to a typical commercial phantom, while the remaining error was ascribed to positioning inaccuracies. While the delivered doses are measured with in‐vivo dosimetry in each fraction, significant underdoses require additional boost fields and can adversely impact treatment outcomes.

While commercial radiotherapy phantoms such as the RANDO phantom are the standard for TSET dosimetry, purpose‐built phantoms have been constructed. Monzari et al. demonstrated the fabrication of a phantom consisting of the head, torso, and leg area, including internal organs.^16^ However, this method does not account for the positioning required in TSET and was suitable only for the reclined technique, which uses a supine position. The constructed phantom offers several advantages over the RANDO phantom, including higher durability, lower cost, and higher slice resolution.

Another approach for dosimetry in TSET is Monte Carlo simulations, which have seen significant development in the simulating of TSET over the last few years. Recent studies include dose comparisons between modified Stanford and rotational techniques.^11^, ^13^ These used computer animation techniques to repose a 3D digital mesh, allowing for testing of any desired position. A study by Nevelsky et al. also demonstrated conversion of RANDO phantom CT scans to a digital model allowing for MC simulations.^17^ Verification measurements taken were consistent with the simulations, proving the feasibility of MC simulation for future personalized treatment QA in TSET.

A preliminary study by Basaglia et al. has demonstrated the suitability of Monte Carlo simulations for full patient‐specific treatment simulation.^18^ The study demonstrated the use of optical scans of patient geometry to create digital tessellated solids for use in Geant4 simulations. This approach enables patient‐specific treatment simulation across the entire body, a capability that was previously unattainable. The full details are set to be published in future papers, so validation and full capabilities are not known yet. However, even with these technologies, physically measuring dose remains critical and advantageous during the commissioning stages.

TSET remains a challenging radiotherapy technique, characterized by large dose variations across the target surface. Given the differences in each individual setup, measuring dose across the body in commission serves as important baseline data. Thus, the development of a 3D‐printed phantom facilitates dose measurements across the body including limbs. This includes areas receiving less than 20% of the prescribed dose, which would necessitate boost fields, and hotspots, which may require shielding. Typically, these adjustments rely on a combination of in‐vivo dosimetry and basic verification measurements using non‐TSET‐specific phantoms during commissioning. The actual doses administered to patients can also significantly deviate from standard phantom measurements, as observed in the study by Evans et al.^8^ To address these challenges, the implementation of a 3D‐printed whole‐body phantom offers potential improvements by enabling more comprehensive measurements across all areas and can accommodate for variations in dose due to differences in patient body size. Consequently, a 3D‐printed phantom not only offers cost‐efficiency compared to commercial general‐use alternatives but also enables a broader range of measurements, which is particularly important in commissioning. The objective of this investigation is to develop such a phantom and utilize it to assess dose distributions during the commissioning stages.

Furthermore, a full‐body phantom could be used to validate new patient‐specific Monte Carlo treatment simulations. Current verifications are mainly limited to dose measurements through the torso and pelvis surfaces, while the dose to limbs cannot be validated with current anthropomorphic phantoms. These areas have more complex geometry than the torso, requiring accurate simulation methods. Thus, the development of a full phantom would enable verification of dose distribution in these areas too.

## 2 METHODS

**FIGURE 1:**
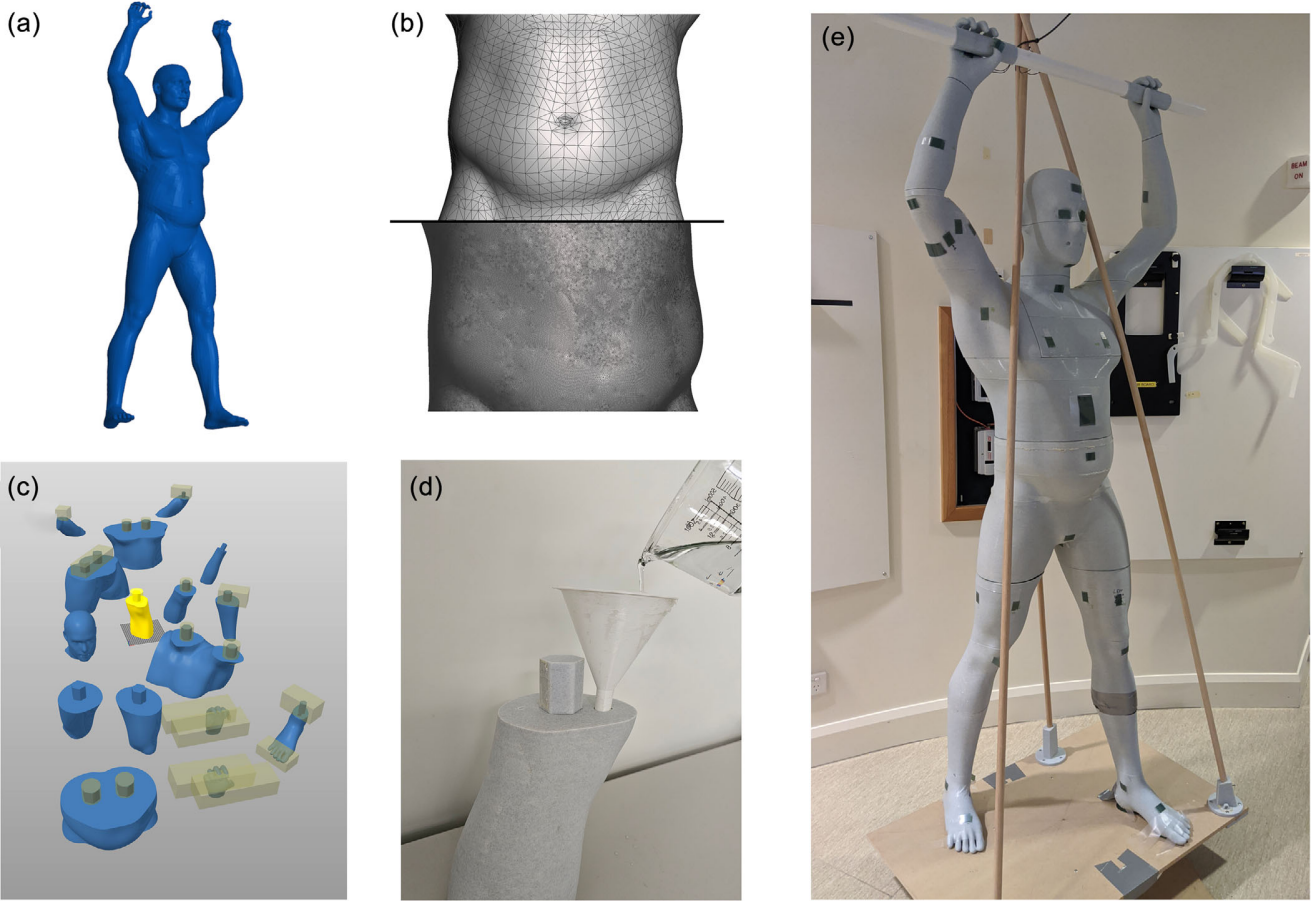
(a) Digital model in MagicPoser, (b) model post‐processing/smoothing, (c) separation into printable parts, (d) filling shells with gelatine, and (e) final constructed phantom.

The process for the phantom creation is shown in Figure 1. (a) The phantom was based on a digital male model and posed in the desired treatment position using MagicPoser (WombatStudio, USA). This model was then scaled to 175 cm height to match the average Australian male height.^19^ (b) This model was then imported into Meshmixer 3.5 (Autodesk, San Francisco, California) for post‐processing, including smoothening and remeshing to increase polygon resolution. (c) The smoothed file was then imported into PrusaSlicer 2.6 (Prusa Research, Prague, Hlavni Mesto Praha, Czech Republic) and segmented into smaller components. Sufficient structural strength was achieved with a 3% gyroid infill, which created a continuous volume for gelatine filling. The shell thickness was set at approximately 2.5 mm. The parts were printed over a total of 20 days, using 20 kg of PLA filament on a Creality CR10‐Max and Prusa MK3S+. (d) The printed shells were filled with tissue‐equivalent gelatine. Gelatine was used due to its cost‐effectiveness, shelf‐life,^20^ and established use in previous theses. (e) To ensure stability during rotation, a wooden structural frame was created using wooden dowels and 3D‐printed mounts.

**FIGURE 2:**
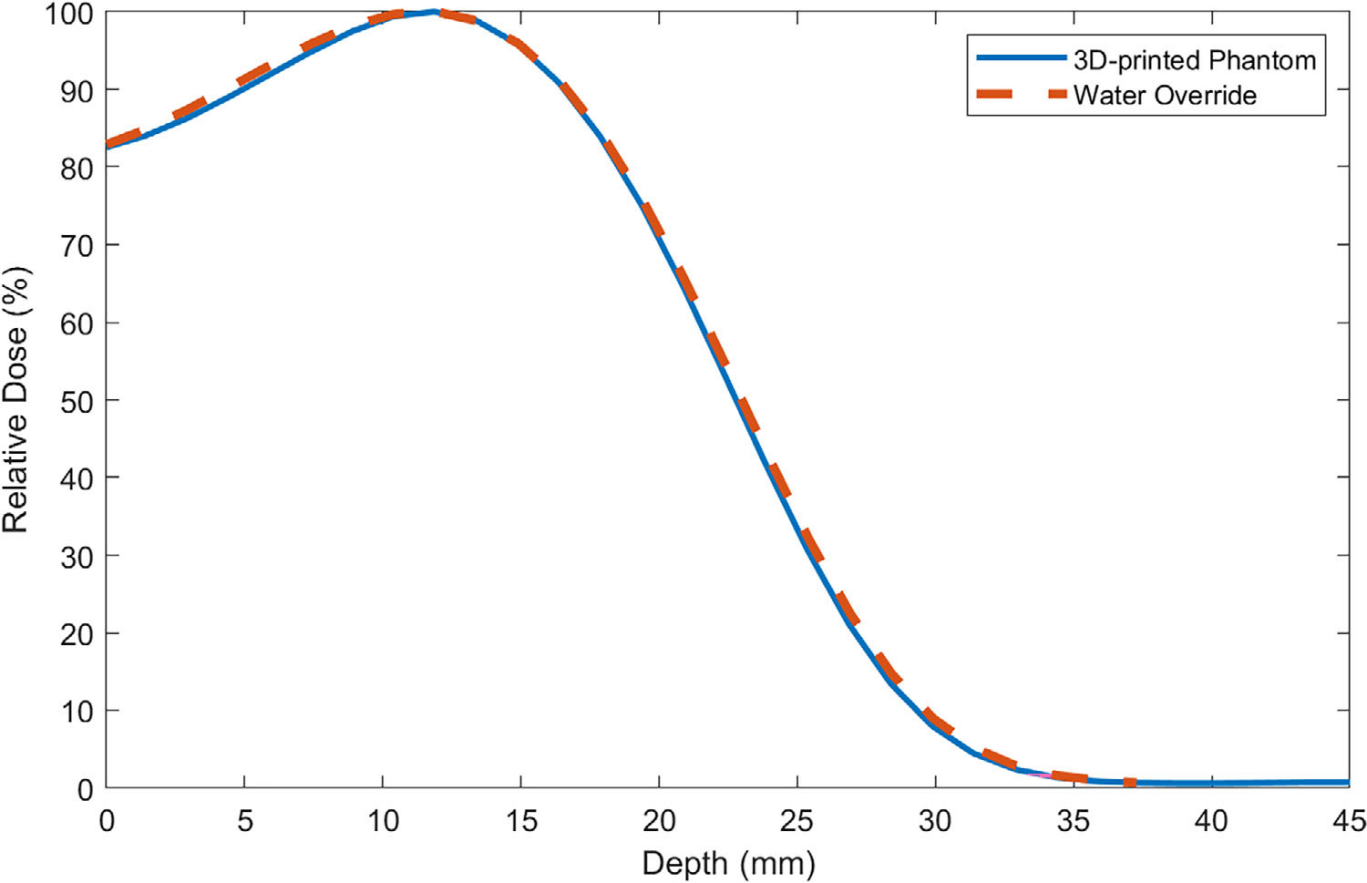
PDDs for the phantom CT scan compared to a water override using Eclipse treatment planning system.

To measure the dosimetric properties of PLA plastic and the phantom, a 15 cm × 15 cm × 5 cm rectangular 3D‐printed phantom was constructed and filled with gelatine. Using the Eclipse treatment planning system (TPS) (Varian Medical Systems, Palo Alto, CA), PDDs through a CT scan of the phantom were compared to the same phantom overrode with water equivalency. This is shown in Figure 2, with a root mean squared error value of 0.9311%. Thus, on average, a real dose would be within 1% of the phantom measurements for soft tissue.

Gafchromic EBT3 film (GAFCHROMIC EBT3; Ashland, Wayne, NJ) was selected due to its ease of use and cost‐effectiveness. Films were calibrated for the 6 MeV electron beam by placing film pieces at d‐max and irradiating with doses from 3 cGy to 300 cGy. Films were then scanned on an Epson Expression 1200XL scanner (Epson America Inc, Long Beach, CA) after developing for 24 h. Noise reduction was performed using a 5×5 median filter using MATLAB. The mean red channel pixel value and standard deviation were calculated in MATLAB from a crop of the central area to exclude the tape used to secure the film. A calibration curve was then fitted to the red channel pixel value using a double exponential equation.

Surface dose was measured at 55 points across the phantom surface using Gafchromic EBT3 film. Films were sized at 2 cm × 3 cm with only the central 1 × 1 cm^2^ area used for measurement to allow space for attachment tape. Measurement points were chosen with clinician advice, while avoiding any seams that could introduce dosimetric inconsistencies. Film was also placed between slices of the phantom, such as in the head and neck regions, to determine the dose at depth for critical structures like the eye and thyroid.

**FIGURE 3:**
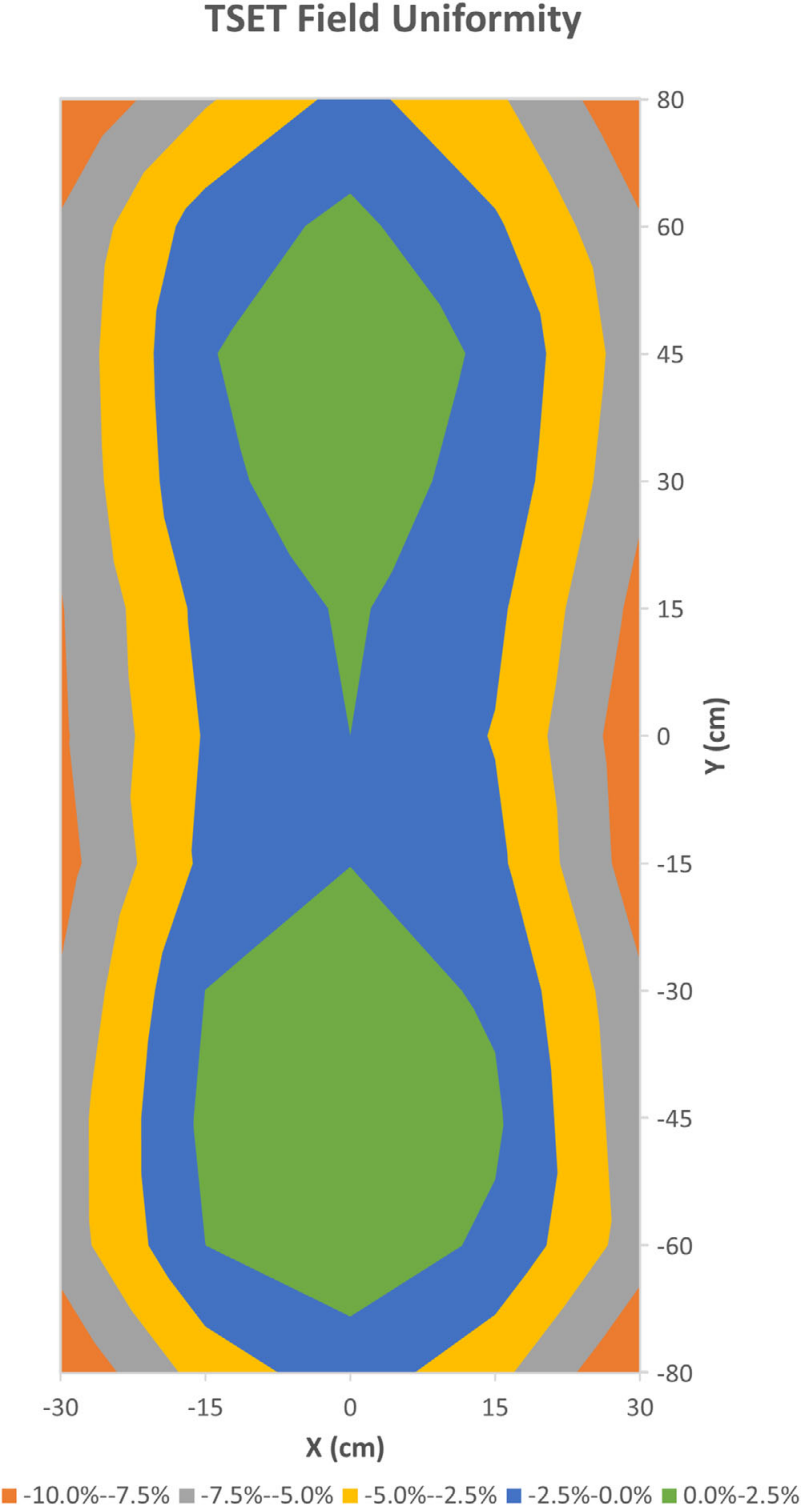
Contour plot of field uniformity at 340 cm SSD.

The phantom was irradiated while under rotation using a Varian TrueBeam linac (Varian Medical Systems Inc., Palo Alto, CA) in 6E HDTSE mode (2500 MU/min) with a collimator size 36 × 36 cm. Following standard procedure, the optimal gantry angle was determined to be ± 18 degrees from the horizontal axis. The corresponding beam uniformity was measured with an FC65‐G ion chamber over 500 MU across a 160 cm × 60 cm area using a 15c cm grid. A plot is shown in Figure 3. The resulting field satisfied the 10% uniformity specified by the EORTC for a 190 × 60 cm area. To perform the rotational technique, a motorized turntable (Pre‐Motion, Ede, Gelderland, the Netherlands) was purchased for this project, with adjustable speed up to 2.5 rpm and a maximum capacity of 200 kg. No degradation filters or scattering foils were employed due to the acceptable field uniformity and percentage depth dose results. A treatment plan was created for a 2 Gy fraction and was calculated with an in‐house spreadsheet based on equations from AAPM TG 30 [2]. This plan prescribed a total of 7168 monitor units across four complete rotations. The reference point used for the prescribed dose was the umbilicus area at an SSD of 340 cm.

## 3 RESULTS

**FIGURE 4:**
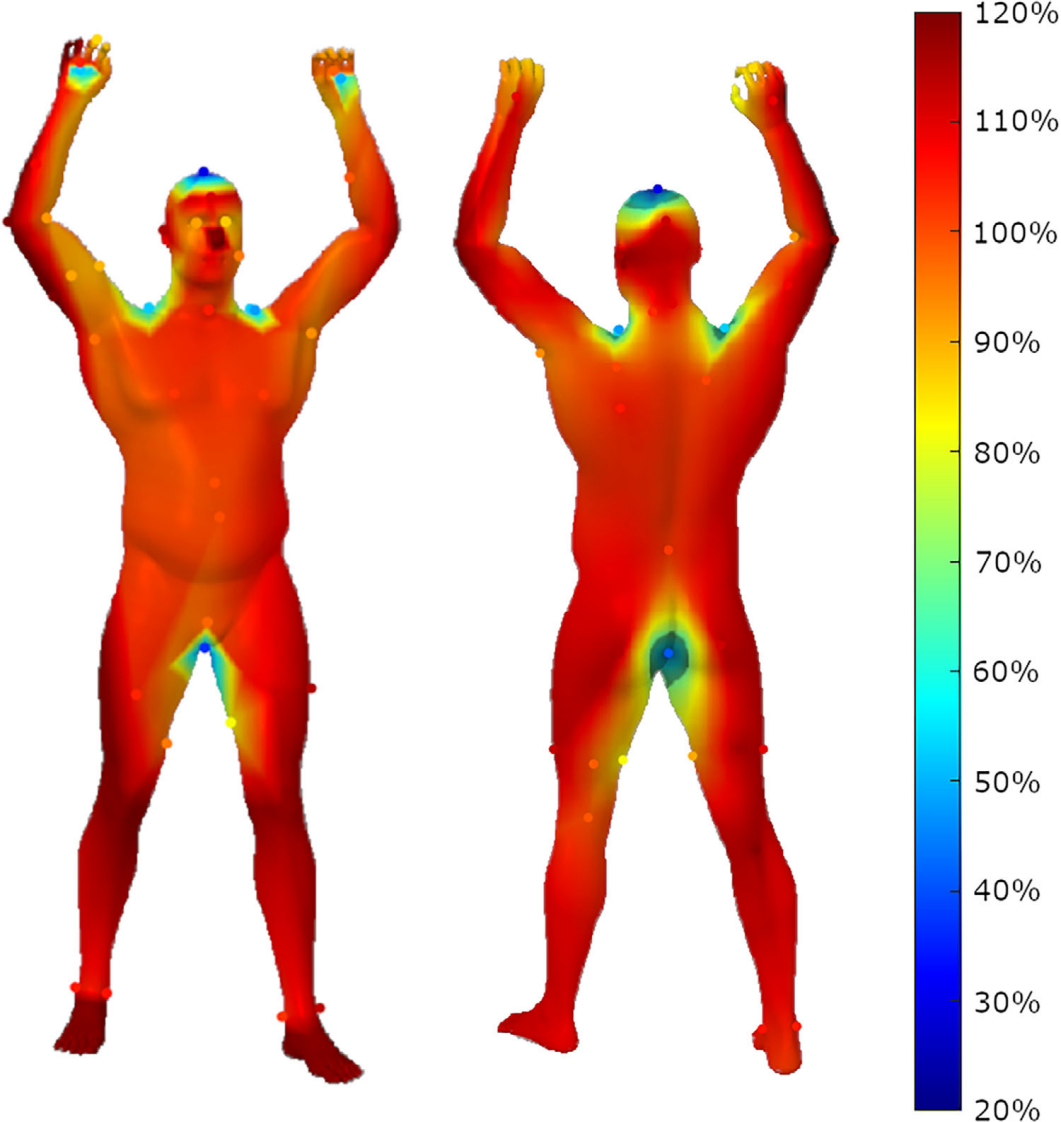
Dose heatmap of phantom results as a percentage of reference dose(%). Measured points are indicated with scatter plot.

A surface dose heatmap was created using the film measurements. This was performed in MATLAB, using natural‐neighbor interpolation to create a 2D matrix that was superimposed on top of the model, as shown in Figure 4. Additionally, dose and pixel area histograms were created using the interpolated data in MATLAB. Notably, this only includes areas visible in Figure 4 and does not include dose to the soles of the feet.

**FIGURE 5:**
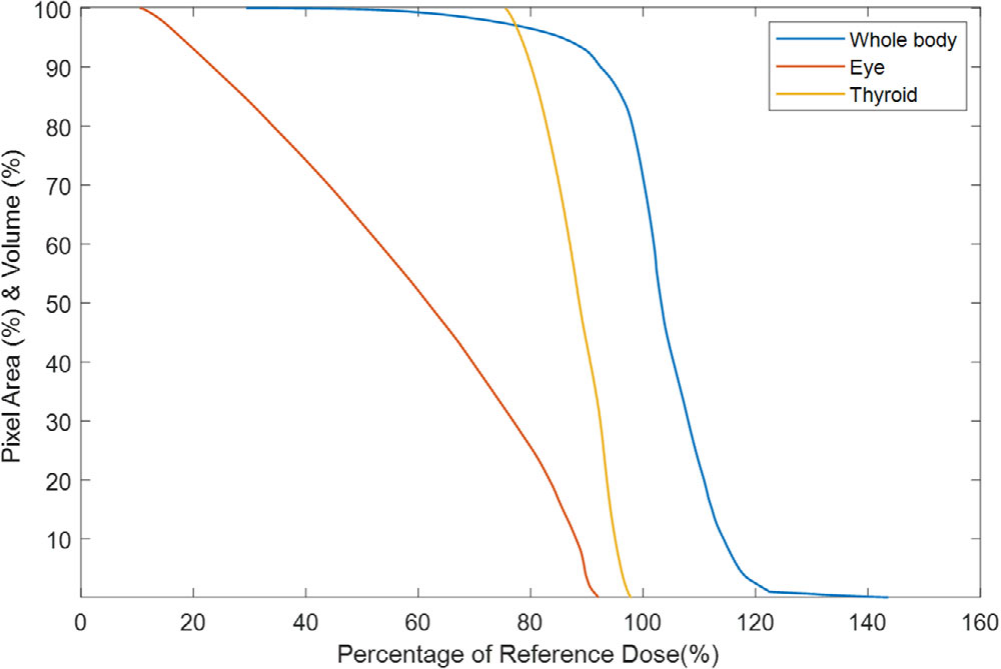
Pixel area histogram illustrating the distribution of radiation dose within the target surface area for the body and volume for eye and thyroid.

Two more dose volume histogram curves were generated from segmentations for the eye and thyroid in Figure 5. Two‐dimensional doses measured on the film were converted to volumetric doses using spherical approximations with assumed uniform vertical doses.

## 4 DISCUSSION

### 4.1 Dose distribution compared to literature

The dose measurements obtained from the phantom, as detailed in Table 1, are largely consistent with expected values, aligning well with the literature with a few positioning and technique differences. The most significant differences can be explained primarily by the TSET technique implemented in this project, specifically the application of a rotational technique compared to those in Table 2, which employ the modified Stanford technique. One notable difference observed was the dose to the axilla which was 97% in the present while the values reported in the literature were 59%,^13^ 60%,^13^ 89%,^21^ and 70%.^22^ All of the previously reported doses were beyond the expected patient variation, with the exception of Kairn et al. with 70% ± 28%.^22^ In rotational techniques, the arms are kept raised throughout the treatment. However, in two out of six positions using the modified Stanford technique, the axilla is shielded from the beam. In addition, the arm positions are lower in the anterior and posterior fields and therefore shield the skin behind them; thus the prescribed dose could not be delivered to those regions. As a result, the measured 97% in the present study met the expectations, and no boost field is required compared to the Stanford technique. This was consistent with the report by Piotrowski et al. on the rotational technique which stated a rotary dose of 104%–107% for the axilla.^23^

**TABLE 1:** Dose distributions in prescribed dose (%) with number of patients in brackets and ± 1 standard deviation.

Site	This study	Antolak *et al.* a.^13^	Antolak *et al.* b.^13^	Baba *et al.* ^21^	Elsayad *et al.* ^14^	Kairn *et al.* ^22^
Scalp Vertex	29 ± 4	76 ± 21 (43)	87 ± 20 (17)	96 ± 4 (6)	98 ± 21 (84)	72 ± 4 (8)
Scalp (top rear)	112 ± 6	‐	105 ± 8 (17)	‐	‐	–
Forehead	111 ± 4	96 ± 8 (27)	‐	98 ± 9 (6)	101 ± 12 (84)	94 ± 3 (8)
Posterior mid‐neck	110 ± 3	103 ± 6 (20)	‐	‐	‐	97 ± 24 (5)
Submental	135 ± 8	101 ± 6 (29)	‐	100 ± 0 (6)	‐	–
Top of Shoulder	50 ± 4	67 ± 15 (103)	74 ± 8 (36)	‐	‐	90 ± 13 (4)
Inner Elbow	92 ± 4	‐	‐	‐	‐	76 ± 20 (5)
Outer Elbow	120 ± 3	‐	90 ± 13 (35)	‐	‐	114 ± 10 (5)
Axilla	97 ± 3	59 ± 19 (108)	60 ± 25 (43)	89 ± 6 (6)	‐	70 ± 28 (5)
Hand (mid dorsum)	109 ± 2	88 ± 7 (85)	85 ± 6 (36)	‐	‐	79 ± 21 (8)
Hand (inner shielded)	51 ± 3	‐	‐	‐	‐	89 ± 30 (8)
Mid‐medial finger	86 ± 10	123 ± 27 (62)	‐	–	‐	–
Upper thorax	102 ± 3	95 ± 9 (33)	93 ± 4 (18)	‐	103 ± 11 (93)	‐
Upper back	105 ± 3	101 ± 6 (16)	93 ± 7 (18)	‐	107 ± 9 (93)	‐
Anterior abdomen	100 ± 3	100 ± 9 (54)	100 ± 4 (21)	101 ± 6 (6)	108 ± 11 (91)	‐
Lower Back	102 ± 3	96 ± 7 (52)	91 ± 7 (19)	‐	105 ± 7 (91)	‐
Mid perineum	37 ± 9	25 ± 21 (34)	‐	‐	‐	16 ± 7 (3)
Mid‐medial thigh	86 ± 4	96 ± 12 (88)	‐	89 ± 15 (6)	90 ± 23 (86)	42 ± 37 (4)
Mid‐anterior thigh	108 ± 3	100 ± 9 (48)	‐	103 ± 2 (6)	‐	‐
Anterior knee	109 ± 3	‐	‐	99 ± 5 (6)	‐	‐
Posterior knee	120 ± 5	‐	‐	93 ± 3 (6)	103 ± 12 (84)	103 ± 12 (4)
Buttock	41 ± 4	‐	58 ± 14 (19)	‐	‐	‐
Foot (mid dorsum)	137 ± 4	124 ± 9 (89)	117 ± 7 (36)	105 ± 5 (6)		116 10 (9)

*Note*: Antolak et al.^13^ (a). was done with a Siemens linear accelerator while (b). was with a Varian accelerator.

**TABLE 2:** TSET implementations for corresponding studies.

	This study	Antolak *et al.* a.^13^	Antolak *et al. b*.^13^	Baba *et* *al*.^21^	Elsayad *et al.* ^14^	Kairn *et al.* ^22^
Linac Type	Varian Truebeam	Siemens Mevatron 80	Varian Clinac 2100C	Clinac DHX	Siemens Primus / Varian Truebeam	‐
Type of detector	Gafchromic EBT3 Film	TLD	TLD	Gafchromic EBT3 Film	TLD	OSLD
Technique	Rotational	Modified Stanford dual field	Modified Stanford dual field	Modified Stanford dual field	Modified Stanford dual field	Modified Stanford dual field

Conversely, while the axilla received a higher dose, the top of the shoulder received a lower dose at 52% of the reference dose. This is a consequence of the higher arm position, with the top of the shoulder at a more oblique angle to the beam. This is also supported by Piotrowski et al., who reported a 55% dose to the top shoulder in a rotational technique.^23^ This is likely to vary significantly with the gantry angles at each setup as well as with patient height.

Another significant difference from the literature is the dose to the mid‐medial hands. In this study, the phantom was holding a suspended bar, simulating the one used for patient stability. As a result, the inner hand acted as a shield, receiving a dose of 51%. The back of the hand received quite a high dose at 109%, and the side of the middle finger received 86%. A high dose is also expected for the top of the fingers when holding the bar but is unable to be measured with film. The hands, thus receive quite a high variation in dose, particularly for our setup with the bar. Due to this high dose variation in our setup, patients will have their hands fully shielded in the commissioned setup, with a boost field delivered afterwards.

Dose to the vertex of the scalp was also extremely underdosed in our setup, receiving only 29% compared to literature values of 76%,^13^ 87%,^13^ 96%,^21^ 98%,^14^ and 72%.^22^ Primarily, the largest factor causing this difference would be the specific setup and height of patients, affecting the angle of electron incidence. An additional factor is the use of film in the study which has a much lower thickness than TLDs. Furthermore, dosimeter positioning is typically only specified as “scalp vertex”, so some differences in dosimeter positioning may exist.

The dose to the mid‐dorsum of the foot was measured at 137% of the reference dose. It is expected that the feet receive a higher surface dose which is consistent with the high‐end of patient dose variation in literature. In the setup used in this study, electrons hitting the mid‐dorsum of the foot will have an estimated incidence angle of around 75°. At this angle of incidence, electrons have a much higher dose at *d*_max_, which occurs at the surface due to increased electron fluence through the central axis.^24^ Particularly for this project, the dose reading is expected to be higher than the literature due to the high vertical uniformity of the field. Furthermore, the presence of the stability frame mounts on the board would cause extra scatter increasing dose readings at the feet.

In comparison, the sole of the foot received a minimum dose of 6% of the reference dose. In the existing literature, the dose for the soles of feet was reported as 34% in standing techniques.^14^ The feet comprise a considerable surface area that is shielded from the radiation beam, resulting in a significantly lower dose reaching the innermost regions of the foot. Thus, the measured dose is heavily dependent on dosimeter positioning, as areas closer to the edge naturally receive a higher dose. Ideally, Gafchromic film can be used in in‐vivo dosimetry to create a comprehensive 2D dose map. Unfortunately, precise film cutting and the influence of tissue elasticity effectively prevent the accurate placement and sizing of film. This region, similarly, is challenging to replicate accurately with a solid 3D‐printed phantom. The presence of the foot arch introduces a high degree of curvature, which can vary significantly depending on the individual's arch height or whether they have flat feet.

### 4.2 Boost areas and areas of concern

Areas requiring a boost dose are determined by a 20% underdose.^25^ Using these criteria, boost fields were determined to be necessary for the top of the shoulders, the soles of the feet, the vertex of the scalp, the perineum, and the hands. This is consistent with literature for the rotational treatment.^23^ Other potential boost sites include areas such as the inframammary folds for pendulous breasts and panniculus in obese patients, which are not measurable with current phantoms.^14^ Given the high variation from patient to patient in many of these areas, this phantom cannot replace in‐vivo dosimetry, although this could potentially be achieved with future personalized phantoms, provided sufficient advancements in 3D‐printing. As previously discussed, it is advisable to shield the hands and administer a boost field to achieve a more uniform dose distribution, particularly as the area is typically characterized by a high dose gradient.

Other areas of concern identified in this study include the mid‐dorsum of the foot and the thumbnail, receiving 137% and 122%, respectively. While the mid‐dorsum of the foot is not particularly radiation sensitive, some shielding should be considered. This can be monitored with in‐vivo dosimetry in earlier fractions, and then shielded for later fractions. Further attention should also be given to the thumbnails and toenails, as the dose was high for the thumbnail (122%) and would likely be high for the toenails based on the mid‐dorsum foot measurement. In this case, shielding for the nails can be considered to avoid nail loss.^26^ Another area that merits consideration for shielding is the male genitalia, although the genitalia was not simulated in our phantom. The adjacent pelvic region received a radiation dose of 97.5%, which can potentially impact the radiosensitive testes. This is consistent with typical TSET side effects, which include potential male infertility.^25^ Therefore, the use of a thick bolus for shielding, along with the inclusion of an accurate testes phantom or attachment for future studies, should be contemplated.

### 4.3 Dose heatmap comparison

The dose heatmap is a novel method of visualizing dose across the body surface in TSET. This is particularly useful for areas of high dose gradient. This is shown in the right elbow, with a slight overdose on the outside, while an underdose was reported for the inner elbow, as seen in Figure 4. Other areas of high dose gradient include the curvature of the shoulder, scalp, and perineum to determine the size of the boost field necessary. Therefore, precise dosimeter placement is crucial in these regions to mitigate errors, or alternatively, additional dosimeters may be strategically positioned to enhance measurement accuracy in these areas.

**FIGURE 6:**
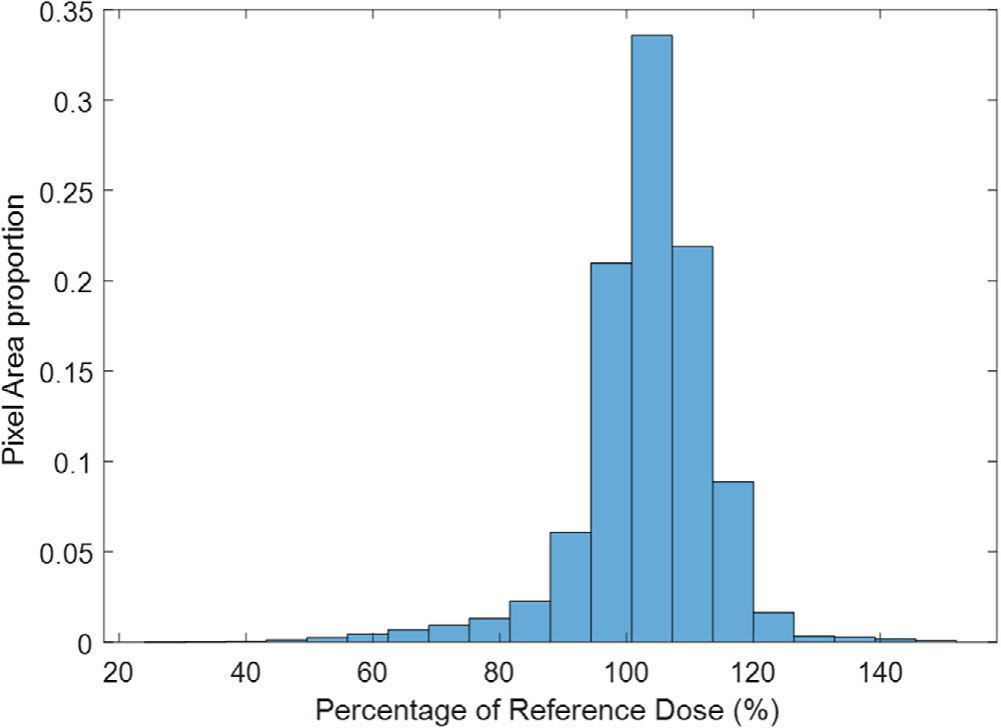
Histogram depicting the distribution of dose pixel values across the dose heatmap.

The interpolated heatmap also allows for quantitative analysis of the dose to the pixels in the image. From Figure 6, approximately 90.8% of the pixels in Figure 4, received a dose within a ± 10% tolerance level. Meanwhile, only 3.5% of pixels received a dose under 80%, and 2.5% received a dose above 120%. Notably, this is pixel area, rather than surface area and does not account for the underdosed feet. Nevertheless, it still serves as an indication of good uniformity of the dose coverage, with low amounts of boost fields and shielding necessary. This analysis can prove a good criterion for evaluating the overall dose distribution.

### 4.4 Phantom comparison

In comparison to conventional slice‐based phantoms such as the RANDO phantom or in Monzari et al.,^16^ the phantom developed in this study lacks significant internal resolution. However, in TSET therapies, organ doses tend to be low due to the steep electron dose falloff at depth. Thus, to improve durability, a section‐based design was used. This phantom focuses on accurate simulation of the intended TSET treatment position. This includes simulation of dose to fine features such as the fingers and toes. As a result, high external resolution can be achieved with accurate measurement of dose shadowing. Simultaneously, measurements of dose at depth were still accomplished between parts but with much lower vertical resolution compared to the slice‐based phantoms.

## 5 CONCLUSION

TSET poses a significant challenge in radiotherapy, with relatively fewer studies and challenges in commissioning and QA. The development of this 3D‐printed phantom was conceived to address this challenge by enabling accurate dose measurements across the entire surface of a patient compared to limited commercial phantoms during the commissioning stage for a rotational technique. Surface dose measurements aligned with existing literature, revealing variations due to the treatment position. This enabled the identification of regions that benefit from dose boosts and areas requiring shielding during commissioning. Heat map analysis also showed dose within a 10% tolerance of the prescribed dose was delivered to over 90% of the visible area in the heat map. Furthermore, suggested shielding, based on our hot spot measurements can enhance the uniformity of dose distribution. The repeatability of the phantom and heatmap informs dosimeter placement to support TSET dose verification. In future studies, this will allow for consistent comparisons between different TSET techniques, while this study focused purely on rotational techniques to expedite commissioning. The establishment of this process with the 3D‐printed phantom could streamline future commissioning and future potential personalized QA procedures.

In conclusion, the 3D‐printed phantom successfully accomplishes the objectives outlined in this investigation. It serves as a whole‐body phantom configured to match the required pose for the rotational TSET technique. To our knowledge, this phantom is the only dedicated method for measuring dose comprehensively across all limbs in a specific TSET position. Additionally, this phantom offers a cost‐effective alternative compared to commercially available counterparts and can be designed to match patient body types and sizes in future situations. Further, this phantom can be used to comprehensively verify Monte Carlo dose planning systems and evaluate different TSET methods.

## AUTHOR CONTRIBUTIONS

Andrew Lee, Zaid Alkhatib, Matthew Fernandez de Viana, Joshua Dass, and Pejman Rowshanfarzad contributed to the conception and design of the study. Andrew Lee, Zaid Alkhatib, MI, Broderick McCallum‐Hee performed measurements, evaluation, and analysis. All authors discussed the results. Joshua Dass provided clinical insights, support from the multidisciplinary lymphoma meetings at SCGH, and presentation/approval at the National Cutaneous Lymphoma Forum of Australia. Andrew Lee wrote the first draft of the manuscript. All authors contributed to the manuscript revision, read, and approved the submitted version.

## CONFLICT OF INTEREST STATEMENT

The authors declare no conflicts of interest.
